# Spatial distribution pattern of mustelids in the eastern edge of the Qinghai–Tibet Plateau

**DOI:** 10.1098/rsos.240294

**Published:** 2024-08-07

**Authors:** Zhuotao Liu, Tengwei Su, Qian Li, Zhaoyuan Li

**Affiliations:** ^1^ Central South University of Forestry and Technology, College of Forestry, Changsha, Hunan 410004, People’s Republic of China; ^2^ Southwest Forest University, College of Soil and Water Conservation, Kunming, Yunnan 650224, People’s Republic of China; ^3^ Yunnan Forestry Technological College, School of Ecological and Environmental Engineering, Kunming, Yunnan 650224, People’s Republic of China; ^4^ Dali University, Institute of Eastern-Himalaya Biodiversity Research, Dali, Yunnan 671000, People’s Republic of China

**Keywords:** Mustelidae, spatial distribution, evolution, species diversity, biogeography, community ecology

## Abstract

Evolutionary theory predicts that the species of an evolutionarily successful taxon would not overlap in spatial distribution. To test the prediction, we document our research on the spatial associations of mustelids, an evolutionarily successful group of order Carnivore, using infrared camera trap data on species distribution collected from the national nature reserves (NNRs) of Liancheng, Wolong, Tangjiahe and Heizhugou in China in 2017–2021. Data showed seven mustelid species occurring in the study area, including *Arctonyx collaris*, *Meles leucurus*, *Martes foina*, *Martes flavigula*, *Mustela altaica*, *Mustela nivalis* and *Mustela sibirica*. Following Ricklef’s definition of biological community, we identified five networks of species associations. The mustelids occurred in the networks. Species from the same genus, such as *M. foina* and *M. flavigula*, stayed in different networks to avoid competition owing to similar feeding habits or habitat preferences. Species with different feeding habits or habitat preferences either occurred in different networks, such as *M. altaica* and *M. flavigula*, or coexisted in the same networks but avoided direct spatial associations, such as *M. foina* and *A. collaris*. Asymmetrical associations were found between different genera, such as *M. foina* and *M. altaica*, or between different subfamilies, such as *M. flavigula* and *A. collaris*. These associations may be attributed to interspecific killing or seed dispersal. However, these associations accounted for only a small proportion and would not impact the species diversity of Mustelidae. It is thus concluded that the prediction is supported by our research findings and that spatial avoidance may be the biogeographic strategy of maintaining the species diversity of the family. We also found that the well protection of the mustelids may benefit the overall biodiversity conservation in Heizhugou, an NNR that has experienced severe deforestation.

## 1. Introduction

Species diversity is not evenly distributed across taxa. Some taxa, such as Mustelidae (order Carnivore) and Bovidae (order Cetartiodactyla), have evolved highly diverse species distribution in various habitats in broad geographical spaces; whereas others, such as Proboscidea, have very few extant species and are distributed in only a few habitat types in small geographical spaces [1]. Marin *et al*. [2] propose an explanation for the differentiated success, suggesting that taxa having experienced longer evolutionary history would have more species than those with shorter evolutionary history. This suggestion is not supported by the fact that Canidae has experienced a much longer evolutionary history than Mustelidae, but its extant species diversity is much poorer than that of Mustelidae [1]. Other studies explore the possible factors influencing species richness in geographical spaces, such as the productivity of biological community, niche growth, population size, habitat heterogeneity, human influence and the dispersing ability of species [3–7], but they show the current state of biological communities and do not explore the reasons of evolutionary background for the differentiated success across taxa.

Species survive and evolve in biological communities. Ecological speciation occurs allopatrically, sympatrically or parapatrically [8], during which different local populations would be faced with differentiated environmental pressure, leading to divergent evolution of the populations (i.e. the accumulation of differences between closely related populations) and speciation [9]. When the newly evolved species encountered [10–12], severe competition would occur owing to their high degree of similarities and thus the overlap of their niches, which could be detrimental to their survival (Gause’s law [12,13]). Therefore, natural selection would favour a taxon whose species avoid spatial association, by which the severe competition would be avoided. This predicts that a successful taxon would have its species occurring in different biological communities. It also predicts that congeneric species would not be spatially associated if occurring in the same communities.

Mustelidae is one of the most successful taxa in the evolution of the order the Carnivore [1]. Its distribution range covers all continents except for Antarctica, Australia, Madagascar and oceanic islands [14], occurring in a wide range of habitats ranging from tundra to tropical rainforests and from high mountains to seas [15]. The family is divided into eight subfamilies (Mustelinae, Melinae, Mellivorinae, Taxidiinae, Ictonychinae, Helictidinae, Guloninae and Lutrinae) [16,17]. Mellivorinae and Taxidiinae may be the earliest subfamilies to diverge (in 16.2 and 15 ma, respectively), followed by Melinae (13.6 ma) [14]. Guloninae diverged in 12.5 ma and Mustelinae in the late Miocene [17–19]. Divergence between genera occurred much later. For example, *Arctonyx* and *Meles* diverged at some 4.28 ma [19] and the Altai weasel (AW) (*Mustela altaica*) and the least weasel (LW) (*Mustela nivalis*) at 2.8 ma [20]. Koepfli *et al.* [16] suggested that the majority of existing mustelids originated in Eurasia and later spread to Africa and the Americas. The species diversity (59 extant species), habitat diversity and distribution range indicate that the Mustelidae is an ideal model taxon for the study to test the above predictions. In an initial attempt to explore the structure of the biological community, we collected data on species distribution using an infrared camera technique in Tangjiahe National Nature Reserve (NNR), Wolong NNR and Heizhugou NNR in Sichuan province and Liancheng NNR in Gansu province near the eastern edge of Qinghai–Tibet Plateau. Following Ricklef’s definition of biological community (which is defined as a network of species interactions [21]), we recognized five association networks in the NNRs. We have also analysed the community environment of giant panda (*Ailuropoda melanoleuca*) [22], snow leopard (*Panthera uncia*) [23] and Sika deer (*Cervus nippon*) [24]. We interpreted the spatial associations into possible interactions between these species and other species. We found that some species which used to be regarded as sympatric in distribution were actually living in different communities and primates lived in predation-free environment [25,26]. We have also made recommendations for their conservation management. Field data show that there are seven mustelids in the study area, including the stone marten (SM) (*Martes foina*), yellow-throated marten (YTM) (*Martes flavigula*), LW (*M. nivalis*), AW (*M. altaica*), Siberian weasel (SW) (*Mustela sibirica*), Asian badger (*Meles leucurus*) and hog badger (*Arctonyx collaris*). In this article, we use the data of these species to test the above predictions, in an attempt to explore the biogeographic mechanism of species diversity maintenance of successful taxa.

## 2. Material and methods

### 2.1. Study area

**Figure 1.**
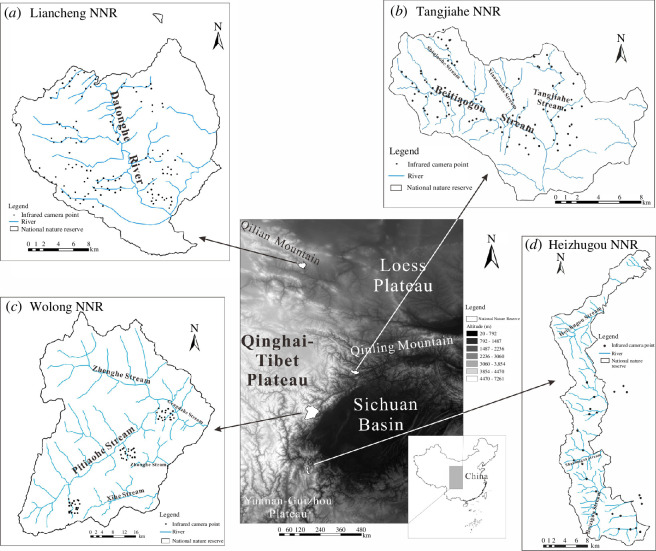
(*a*–*d*) The geographic location of the study area and infrared camera sites in the four NNRs. (Note: the black points mean infrared camera points. The blue lines mean rivers or streams. The black lines mean national nature reserve’s boundaries.)

The study area covers four NNRs in the eastern edge of the Qinghai–Tibet Plateau. The NNRs represent different major topographical types: Liancheng NNR in the southeast of the Qilian Mountains near the Loess Plateau (102°26′−102°55′E, 36°33′−36°48′N, Gansu province), Tangjiahe NNR on the southwestern slope of the Qinling Mountain Range (104°36′−104°52′E, 32°30′−32°41′N, Sichuan province), Wolong NNR in the eastern part of the Qinghai–Tibet Plateau (102°52′−103°24′E, 30°45′−31°25′N, Sichuan) and Heizhugou NNR on the Yunnan–Guizhou Plateau (102°54′29″−103°04′07″E, 28°39′54″−29°08′54″N, Sichuan; figure 1). Liancheng NNR (altitude ranging from 1870 to 3616 m a.s.l., with a difference of 1746 m), Tangjiahe NNR (1150 to 3864 m a.s.l., difference of 2714 m) and Heizhugou NNR (1054 to 4288 m a.s.l., difference of 3234 m) are relatively gentler than Wolong NNR (1150 to 6250 m a.s.l., difference of 5100 m) in terrain [27–30]. The terrain in Wolong NNR fluctuates from the low altitudes in the southwest to the high altitudes in the northwest. Steep valleys fragment the plateau surface into three big pieces and many small pieces. Each piece is surrounded by upward and downward cliffs as high as 300 m. The east-westward Qinling Mountain range blocks the humid air current from the tropical Pacific. It is thus colder and drier in Liancheng NNR in the north to the Qinling (with an annual average temperature of 7.4°C, annual precipitation of 419 mm [27]) than in Tangjiahe NNR (12°C, 1000–1300 mm [28]), Wolong NNR (8.9°C, 800–1000 mm [29]) and Heizhugou NNR (16°C, 1900–2000 mm [30]) in the south.

Wolong, Heizhugou and Tangjiahe NNRs are located in the Indomalayan Realm in zoogeography, and Liancheng is located in the Palearctic Realm [31]. Deep valleys are full of warm air currents from the south, which supports Indomalayan species to disperse northward to reach the study area. High altitudes provide passageways for Palearctic species to disperse southward to reach the area. Native species which evolved in the Pleistocene further enrich the biodiversity of the area. As a result, this area has become a biodiversity hotspot with international significance [32]. Diverse environments differentiated during Himalayan orogeny and Pleistocene glaciers have made the area an ideal place for biogeographic and ecological evolutionary research (figure 1).

### 2.2. Field data collection

ArcGIS was used to generate a system of 1 × 1 km grids to cover the map of the four NNRs. A total of ≥5% of accessible grid cells were randomly selected, and each cell was installed with an infrared camera (models: Yianws L710 and Ltl Acorn 6210). The mountain terrain deters our fieldworkers from accessing some areas, including valleys, mountain peaks and the places that the fieldworkers would have to hike for ≥3 days from our camping sites. When a selected cell was located in such an inaccessible places, we abandoned it and re-selected a new cell. As a result, there were many more abandoned cells (and thus re-selected cells) in Wolong than in the other three NNRs, and the camera sites looked unevenly distributed on two-dimensional maps (figure 1). Ovital Map was used to navigate our field workers to get into the cells to install cameras. The distance between neighbouring cameras was maintained at ≥300 m. Cameras were placed in the major habitat types of the grids and fixed on tree trunks or other objects 50–80 cm above the ground, facing parallel to the ground. The angle of the lens allowed the cameras to trap images of animals on the ground in the front scenery and the animals on scrubs and middle canopy in the middle and distant sceneries; therefore, we obtained information on terrestrial and semi-arboreal species. Batteries and memory cards were replaced once every three months. Infrared cameras are triggered by the movement of infrared rays; thus, ectothermic animals do not trigger cameras. Therefore, we obtained thermostatic animals including birds and mammals. The coordinates and altitudes were measured using a compass on the mobile phones. Slopes and aspects were measured using the Ovital Map app on the phones. The height of the vegetation and the canopy coverage were estimated visually.

Owing to the different habits of the species, all cameras worked for ≥12 months to obtain complete data on the distribution of the species. There were 128 sites randomly selected in Liancheng NNR, with cameras continuously working from July 2018 to June 2020; 103 sites in Tangjiahe NNR, with cameras working from September 2019 to December 2020; and 60 sites in Wolong NNR, with cameras working from February 2017 to April 2018. The northern part of the Heizhugou NNR was inaccessible, so data were collected in the central and southern parts with 24 cameras working from September 2019 to December 2020. We checked the images and videos and identified the species with reference to Smith & Xie [33] and Mackinnon *et al.* [34]. Murids were identified based on the dental morphological features. Consequently, our technique did not support identification, so murids were not identified and we put all murids into a single form, ‘murids’.

Considering that solitary animals and social animals have different ranging behaviours, resulting in different correlations between population density and occurrence frequency at a given site, and that some images with poor definition, especially taken at night, were not good enough to identify individuals, we only recorded information about the presence or absence of a species at a site, ignoring its occurrence frequency. Therefore, the data type is dichotomous. We then counted the number of cameras in which (a) both species in question were present, (b) only one species was present, (c) only the other species was present, and (d) both species were absent. According to Siegel & Castellan [35], we used the data to calculate the Phi coefficients *r*_ø_ and the Lambda statistics *L*_*B*_.

### 2.3. Measurement of spatial associations: the Phi coefficient *r_ø_*

In traditional ecological literature, ecological relations are mostly studied based on occasional observations. These observations may be caused by random factors and therefore may not indicate regular interspecific interactions [36]. Forbes pioneered the use of spatial association as a currency to measure ecological relationships between species, but most studies since then contained associations caused by random factors [37]. To rule out such associations, we used the Phi coefficient *r*_ø_ which is a measure of the degree of association between two sets of attributes measured on a dichotomous scale. The calculation was carried out as follows [35].


(2.1) $$r_{\emptyset }=\frac{\left|ad-bc\right|}{\sqrt{\left(a+b)(b+c)(a+c)(b+d\right)}},$$

where the *r*_*ø*_ was Phi coefficient and measured the strength of association between the species in question. The absolute value of *r*_*ø*_ ranged from 0 to 1. The association was positive ‘+’ if the value of ‘*ad − bc*’ was greater than zero, meaning the two species attract together; and it was negative ‘−’ if the value was smaller than zero, meaning the two species avoid or push each other apart. The meaning of ‘*a*’, ‘*b*’, ‘*c*’ and ‘*d*’ was the number of cameras defined above.

The coefficient may be caused by any kind of co-occurrences of two species, including those owing to random factors. Thus, the second step is to calculate *χ^2^* for testing the significance of the calculated *r*_ø_, which is as follows:


(2.2) $$\chi^{2}=\frac{N{\left(\left|ad-bc\right|-0.5N\right)}^{2}}{\left(a+b)(b+c)(a+c)(b+d\right)},$$

where the ‘*N*’ was the total number of cameras, i.e. *N = a + b + c + d*. When *χ^2^* < 3.841, *p* > 0.05, *r_ø_* was not statistically significant, implying that the coefficient was caused by random factors and the association was not supported by ecological interaction, we abandoned the coefficient. The *r*_*ø*_ was significant when *χ^2^* ≥ 3.841, *p* ≤ 0.05 [35], indicating the species of the pair regularly co-occurred at the same sites owing to their ecological interaction, and thus, we retained the pair.

### 2.4. Species network assembly

We used the retained positive associations as bonds to connect species into species networks using NetDraw^®^ (edition 2.148).

### 2.5. Testing the properties of associations: the Lambda *L_B_* test statistics

Species interactions are classified into five categories [21]. The first category is that both species in a pair benefit from the interaction. This category includes mutualism, in which the populations of the species tend to coexist within each other’s range. The spatial association is bi-directionally or mutually asymmetric. The second category is that only one of the species benefits from the interaction, while the other neither benefits nor suffers. This category includes commensalism, in which the population of the benefiting species tends to occur within the spatial distribution range of the species providing the benefit. Thus, the spatial association is uni-directionally asymmetric. The third category involves one species benefiting from the association while the other suffers. This category includes predator–prey, herbivore–plant and parasite–host interactions. The species that benefit would tend to occur within the range of the species that are suffering. The association is uni-directionally asymmetric, similar to the second category in spatial relationships. The fourth category involves both species suffering from the interaction, which includes competition. In such interactions, if a species gives up the competition and moves away, it would lose more in a saturated community. Thus, it would be beneficial to continue competing regardless of whether other species remain, and the association is symmetrical. The last category includes amensalism, in which one species suffers from the interaction while the other neither suffers nor benefits. The suffering species would avoid spatial overlaps with the others, resulting in no spatial association. Consequently, such an interaction is incidentally observed in the community [21]. Therefore, we tested whether an association was mutually asymmetrical, uni-directionally asymmetrical or symmetrical to further explore the types of interactions (categories 1–4) underlying the retained species associations. Considering the dichotomous data type in this research, we used the Lambda statistics [35].

The first step of the calculation was to set up a contingency table as follows (table 1).

**Table 1.** Contingency table for the Lambda statistics.

species A species B	*A*_1_	*A*_2_	total
*B*_1_	*n*_11_	*n*_12_	*R*_1_
*B*_2_	*n*_21_	*n*_22_	*R*_2_
total	*C*_1_	*C*_2_	*N*

Here, ‘*A*_1_’ was the variable of species A present and ‘*A*_2_’ the variable of the species when absent. Similarly, ‘*B*_1_’ was the variable of species B present and ‘*B*_2_’ was the variable of the species when absent. Accordingly, ‘*n*_11_’ was the number of cameras in which both species were present and ‘*n*_22_’ was the number of cameras in which both species were absent. ‘*n*_12_’ and ‘*n*_21_’ were the numbers of cameras in which only one species was present and the other absent. ‘*R_i_* (*i* = 1, 2)’ and ‘*C*_*j*_ (*j* = 1, 2)’ were the total number of the respective columns and rows. ‘*N*’ was the total number of cameras.

The second step was to carry out the following calculations.


(2.3) $$L_{B}=\frac{\sum_{j=1}^{2}n_{Mj}-\max\;\left(R_{i}\right)}{N-\max\;\left(R_{i}\right)},$$

where ‘*n*_Mj_’ was the maximum number in column *j* and ‘max(*R*_*i*_)’ the maximum sum of the rows. ‘*L*_*B*_’ was the measurement for the degree to which the presence of species A predicted the presence of species B. For a given pair of species X and Y, when *L*_*B*_ was used to measure the degree of species X to predict species Y (i.e. X at the place for species A in the contingency table and Y at the place for species B), *L*_*A*_ would be used to measure the degree of Y to predict the presence of X. *L*_*B*_ could be different from *L*_*A*_.

The significance of the calculated statistics *L_B_* was tested using the following calculation.


(2.4) $$\text{var}\left(L_{B}\right)=\frac{\left(N-\sum_{j=\text{1}}^{2}n_{Mj}\right)\left(\sum_{j=\text{1}}^{2}n_{Mj}+\max\;\left(R_{j}\right)-2\sum^{\prime }n_{Mj}\right)}{{\;}^{{\left[N-\max\;\left(R_{j}\right)\right]}^{3}}},$$

here *∑′n_Mj_* is the sum of max column numbers in the row that max(*R_i_*) locates. In this study, there was only one max column number in the row, so *∑′n_Mj_ = n_Mj_*.

The third step was to carry out the following calculation.


(2.5) $$\lambda_{B0}={\text{L}}_{B}-{\text{Z}}_{p=0.05}\times \sqrt{\text{var}\left(L_{B}\right)},$$

where ‘*λ*_*B0*_’ was the predictability value of species A to species B with statistical significance, and *Z_p_*_= 0.05_ was available from the Lambda statistic table (table 1) [35]. The calculated *L_B_* was significant when *L_B_* > *λ_B_*_0_. The values of *λ_B_*_0_ and *L_B_* were used, respectively, as the lower and upper limits of the predictability of species A to species B, and the likelihood was calculated as follows.

The likelihood of the lower limit = *λ_B0_* × 100%

The likelihood of the upper limit = *L_B_* × 100%

Similar tests were carried out for the significance of *L_A_*.

## 3. Results

### 3.1. Fauna

A total of 117 974 images and videos were obtained in the four NNRs, from which 62 species were identified, plus unidentified murids, belonging to 52 genera from 20 families of six orders, including one bird order (Galliformes) and five mammal orders (Cetartiodactyla, Carnivora, Primates, Lagomorpha and Rodentia). Seven mustelids were found, including the SM (*Martes foina*), YTM (*M. flavigula*), LW (*Mustela nivalis*), AW (*M. altaica*) and SW (*M. sibirica*) from Mustelinae and Asian badger (*Meles leucurus*) and hog badger (*A. collaris*) from Melinae. The richness of mustelids was similar between the four NNRs, but species identity was different. SWs were found in all of the NNRs. YTMs and hog badgers were found in the Tangjiahe, Wolong and Heizhugou NNRs. SMs and AWs were found in the Liancheng and Wolong NNRs. The LWs were found in the Tangjiahe NNR and Asian badgers in the Liancheng NNR only. There were 21 potential pairs between the mustelids (table 2).

**Table 2.** Potential species pairs of mustelids and their Phi coefficients. *p* ≤ 0.05 for all values in the table. ‘–’ means no direct association. Distribution pattern：palaearctic type (U), oriental type (W) and others (O).

mustelids in different networks mustelids	Heizhugou		Wolong LAN	Wolong HAN			Tangjiahe LAN		Tangjiahe HAN		Liancheng		
	hog badger (*A. collaris*) (W)	yellow-throated marten (*M. flavigula*) (W)	yellow-throated marten (*M. flavigula*) (W)	stone marten (*M. foina*) (U)	Altai weasel (*M. altaica*) (O)	hog badger (*A. collaris*) (W)	hog badger (*A. collaris*) (W)	yellow-throated marten (*M. flavigula*) (W)	Siberian weasel (*M. sibirica*) (U)	least weasel (*M. nivalis*) (U)	Asian badger (*M. leucurus*) (U)	stone marten (*M. foina*) (U)	Altai weasel (*M. altaica*) (O)
Altai weasel (*M. altaica*) (O)	—	—	—	0.62	—	—	—	—	—	—	—	—	—
yellow-throated marten (*M. flavigula*) (W)	—	—	—	—	—	—	0.42	—	—	—	—	—	—
Asian badger (*M. leucurus*) (U)	—	—	—	—	—	—	—	—	—	—	—	—	—
least weasel (*M. nivalis*) (U)	—	—	—	—	—	—	—	—	—	—	—	—	—
Siberian weasel (*M. sibirica*) (U)	—	—	—	—	—	—	—	—	—	—	—	—	—
stone marten (*M. foina*) (U)	—	—	—	—	0.62	—	—	—	—	—	—	—	—
hog badger (*A. collaris*) (W)	—	—	—	—	—	—	—	0.42	—	—	—	—	—

### 3.2. Species networks

**Figure 2.**
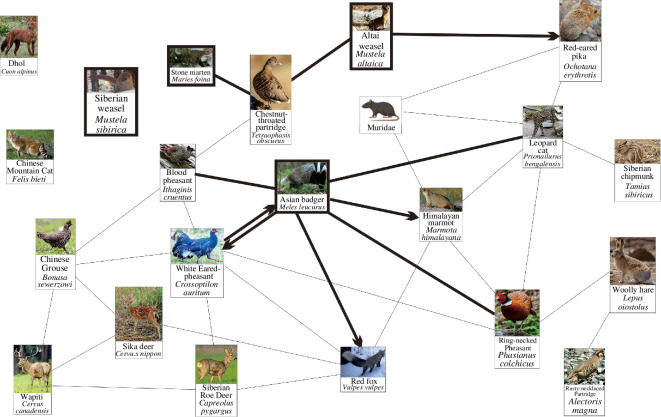
Spatial associations of mustelids in Liancheng NNR (Note: single arrows indicate uni-directional asymmetric associations, double arrows indicate bi-directional asymmetric associations and no-arrow lines indicate symmetric associations. Images come from the infrared camera data of this research).

A total of 22 species were identified within Liancheng NNR, of which 19 could be interwoven into a network (figure 2). The network ranged in altitude from 1977 to 3126 m a.s.l. where the habitats were dominated by shrubs and coniferous forests. The Asian badger, AW and SM were found in the network. SMs appeared in deciduous broadleaf forests, broadleaf–coniferous mixed forests and coniferous forests at 2111–2993 m a.s.l.. AWs were found in deciduous broadleaf forests, broadleaf–coniferous mixed forests, coniferous forests and shrubs at 2022–3026 m a.s.l., and Asian badgers inhabited broadleaf–coniferous mixed forests, coniferous forests and shrubs at 1997–3041 m a.s.l.

**Figure 3.**
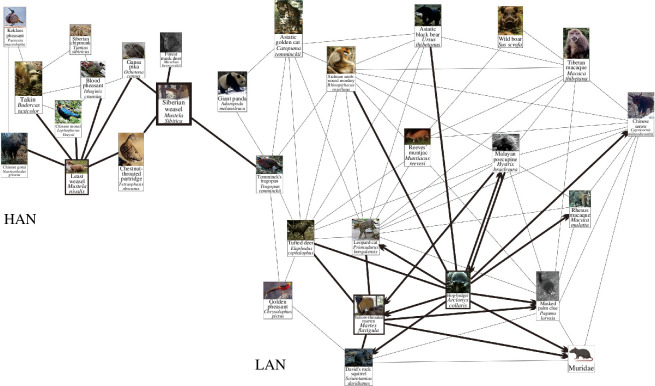
Spatial associations of mustelids in Tangjiahe NNR (Note: single arrows indicate uni-directional asymmetric associations, double arrows indicate bi-directional asymmetric associations and no-arrow lines indicate symmetric associations. Images come from the infrared camera data of this research).

A total of 29 species plus unidentified murids were found in Tangjiahe NNR, and these species were interwoven into two networks (figure 3). The first network, called Tangjiahe high-altitude network (HAN), was composed of 11 species, located at 2865–3373 m a.s.l. where the habitats were dominated by shrub meadows and coniferous forests. LWs and SWs occurred in this network. LWs appeared in shrubs at 3041 m a.s.l. and SWs in deciduous broadleaf forests and broadleaf–coniferous mixed forests at 1256–3041 m a.s.l. The second network, called Tangjiahe low-altitude network (LAN), was composed of 19 species, located at 1220–2759 m a.s.l. where the habitats were dominated by broadleaf–coniferous mixed forests. YTMs and hog badgers were found in this network. Both species were found in deciduous broadleaf forests and broadleaf–coniferous mixed forests but at different altitudes (YTMs at 1220–2397 m a.s.l. and hog badgers at 1256–3041 m a.s.l.). The two networks were spatially linked by an association between the SW and the Temminck’s tragopan.

**Figure 4.**
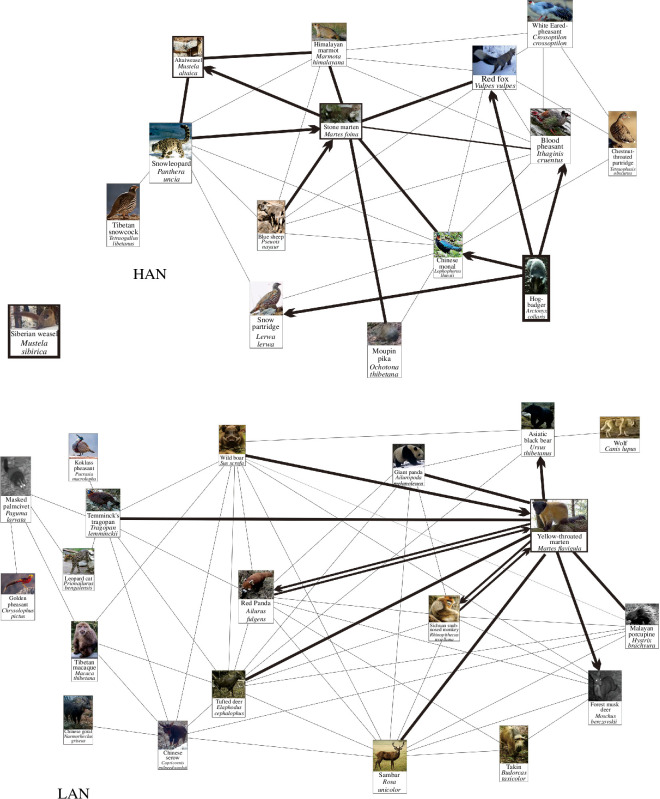
Spatial associations of mustelids in Wolong NNR (Note: single arrows indicate uni-directional asymmetric associations, double arrows indicate bi-directional asymmetric associations and no-arrow lines indicate symmetric associations. Images come from the infrared camera data of this research).

A total of 35 species were found in Wolong NNR, and these species were interwoven into two networks (figure 4). The first network, called Wolong HAN, was composed of 14 species, located at 3718–4430 m a.s.l. where the habitats were dominated by shrub meadows and screes. SMs, AWs and hog badgers occurred in this network. SMs appeared on shrub meadows and screes at 4052–4338 m a.s.l. and AWs on screes at 4303–4338 m a.s.l. Hog badgers were found in broadleaf–coniferous mixed forests, shrubs and on screes at 2473–4430 m a.s.l. The second network, called Wolong LAN, consisted of 20 species, located at 1749–3361 m a.s.l. where the habitats were dominated by broadleaf–coniferous mixed forests. YTMs occurred in this network, inhabiting broadleaf–coniferous mixed forests at 1979–3361 m a.s.l. SWs remained outside the Wolong networks.

**Figure 5.**
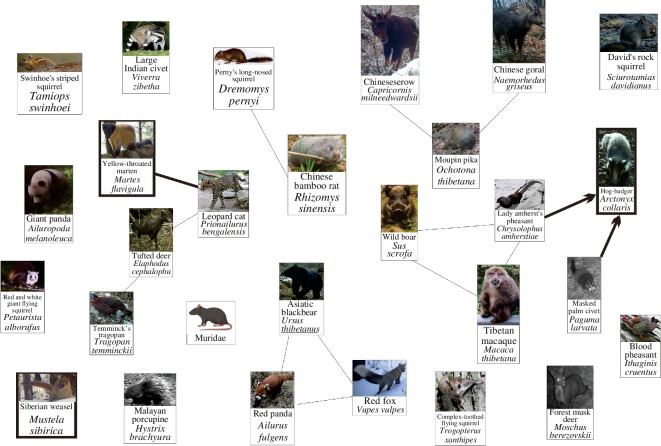
Spatial associations of mustelids in Heizhugou NNR (Note: single arrows indicate uni-directional asymmetric associations and no-arrow lines indicate symmetric associations. Images come from the infrared camera data of this research).

A total of 28 species were recorded in the Heizhugou NNR (figure 5). These species did not appear in the networks; however, five species groups were found. Three mustelids were found, of which the hog badger was associated with the masked palm civet, Lady amherst’s pheasant and the YTM with the leopard cat. Again, the SW was not associated with any other species.

### 3.3. Spatial associations of mustelids

#### 3.3.1. The stone marten and Altai weasel

Two mustelids appeared in the Liancheng network and Wolong HAN. In Wolong HAN, SMs were directly and asymmetrically associated with the AW, in which SMs tended to occur in the range of the AW (table 3). They appeared in screes with a slope of 21−40° at 4303–4338 m a.s.l. However, in the Liancheng network, they were not directly associated with each other. The SM was associated with the chestnut-throated partridge (*Tetraophasis obscures*) only, whereas the AW was further associated with the red-eared pika (*Ochotona erythrotis*) in addition to the chestnut-throated partridge. The spatial associations of the two mustelids was much more complicated in Wolong HAN than in Liancheng network. In addition to the asymmetric association between them, the AW was associated with two other species and the SM with seven other species.

**Table 3.** Spatial associations of *M. foina* and *M. Altaica*. *p* ≤ 0.05 for all values in the table；distribution pattern：palaearctic type (U), oriental type (W) and others (O). ‘–’ means no asymmetrical. ‘A’ represents the prediction of the associated species to the corresponding mustelids, and ‘B’ represents the prediction of the corresponding mustelids to the associated species.

network/NNR	species pair	the number of occurrence sites of mustelids	the number of occurrence sites of related species	the number of occurrence sites	Phi coefficients (*r*_*ø*_)	*L_A_* (upper)	*λ_A_* (lower)	*L_B_* (upper)	*λ_B_* (lower）
Wolong HAN	stone marten (*M. foina*)–blood pheasant (*Ithaginis cruentus*)	5/60	16/60	4	0.36	—	—	—	—
Wolong HAN	stone marten (*M. foina*)–Chinese monal (*Lophophorus lhuysii*)	5/60	15/60	4	0.38	—	—	—	—
Wolong HAN	stone marten (*M. foina*)–snow leopard (*P. uncia*)	5/60	8/60	4	0.59	—	—	0.38	0.01
Wolong HAN	stone marten (*M. foina*)–red fox (*Vulpes vulpes*)	5/60	12/60	4	0.45	—	—	—	—
Wolong HAN	stone marten (*M. foina*)–Altai weasel (*M. altaica*)	5/60	2/60	2	0.62	0.4	0.04	—	—
Wolong HAN	stone marten (*M. foina*)–Himalayan marmot (*Marmota himalayana*)	5/60	8/60	3	0.41	—	—	—	—
Wolong HAN	stone marten (*M. foina*)–blue sheep (*Pseudois nayaur*)	5/60	13/60	5	0.48	—	—	0.38	0.16
Wolong HAN	stone marten (*M. foina*)–Moupin pika (*Ochotona thibetana*)	5/60	3/60	2	0.57	—	—	—	—
Liancheng	stone marten (*M. foina*)–chestnut-throated partridge (*Tetraophasis obscurus*)	29/128	14/128	8	0.29	—	—	—	—
Liancheng	Altai weasel (*M. altaica*)–chestnut-throated partridge (*T. obscurus*)	10/128	14/128	4	0.27	—	—	—	—
Liancheng	Altai weasel (*M. altaica*)–red-eared pika (*O. erythrotis*)	10/128	16/128	4	0.24	—	—	—	—
Wolong HAN	Altai weasel (*M. altaica*)–snow leopard (*P. uncia*)	2/60	8/60	2	0.47	—	—	—	—
Wolong HAN	Altai weasel (*M. altaica*)–Himalayan marmot (*M. himalayana*)	2/60	8/60	2	0.47	—	—	—	—

#### 3.3.2. The least weasel

The LW appeared in Tangjiahe HAN, and no mustelids were associated with it (table 4). The species appeared in shrub meadows with a slope of 6−20° at 3041 m a.s.l. The SW was also found in Tangjiahe HAN and appeared in broadleaf−coniferous mixed forests, deciduous broadleaf forests and shrub meadows with a slope of 6−30° at 1805–3041 m a.s.l. The two mustelids diverged in habitat use and were not directly associated in Tangjiahe HAN (figure 3). For details on the SW, refer to §3.3.5.

**Table 4.** Spatial associations of *M. nivalis* (See the caption in table 3).

network/NNR	species pair	the number of occurrence sites of Mustelids	the number of occurrence sites of related species	the number of occurrence sites	Phi coefficients (*r*_*ø*_)	*L_A_* (upper）	*λ_A_* (lower）	*L_B_* (upper）	*λ_B_* (lower）
Tangjiahe HAN	least weasel (*M. nivalis*)–chestnut-throated partridge (*T. obscurus*)	1/103	3/103	1	0.57	—	—	—	—
Tangjiahe HAN	last weasel (*M. nivalis*)–blood pheasant (*I. cruentus*)	1/103	2/103	1	0.7	—	—	—	—
Tangjiahe HAN	least weasel (*M. nivalis*)–Chinese monal (*L. lhuysii*)	1/103	2/103	1	0.7	—	—	—	—
Tangjiahe HAN	least weasel (*M. nivalis*)–Gansu pika (*Ochotona cansus*)	1/103	3/103	1	0.57	—	—	—	—
Tangjiahe HAN	least weasel (*M. nivalis*)–Chinese goral (*Naemorhedus griseu*)	1/103	99/103	1	0.02	—	—	—	—
Tangjiahe HAN	least weasel (*M. nivalis*)–Takin (*Budorcas taxicolor*)	1/103	101/103	1	0.02	—	—	—	—

#### 3.3.3. The Asian badger

The Asian badger was found only in Liancheng NNR, with uni-directionally asymmetrical associations with six species, but none of the associated species were mustelids (table 5).

**Table 5.** Spatial associations of *M. leucurus* (refer the caption in table 3).

network/NNR	species pair	the number of occurrence sites of Mustelids	the number of occurrence sites of related species	the number of occurrence sites	Phi coefficients (*r*_*ø*_)	*L_A_* (upper)	*λ_A_* (lower)	*L_B_* (upper)	*λ_B_* (lower）
Liancheng	Asian badger (*M. leucurus*)–blood pheasant (*I. cruentus*)	72/128	27/128	24	0.34	—	—	—	—
Liancheng	Asian badger (*M. leucurus*)–common pheasant (*L. lhuysi*)	72/128	41/128	30	0.23	—	—	—	—
Liancheng	Asian badger (*M. leucurus*) blue eared-pheasant (*Crossoptilon auritum*)	72/128	71/128	55	0.48	0.41	0.24	0.42	0.26
Liancheng	Asian badger (*M. leucurus*)–red fox (*V. vulpes*)	72/128	98/128	66	0.3	0.32	0.19	—	—
Liancheng	Asian badger (*M. leucurus*) leopard cat (*Prionailurus bengalensis*)	72/128	33/128	27	0.4	—	—	—	—
Liancheng	Asian badger (*M. leucurus*)–Himalayan marmot (*M. himalayana*)	72/128	86/128	58	0.32	0.25	0.08	—	—

#### 3.3.4. The yellow-throated marten and hog badger

The two mustelids appeared in Tangjiahe LAN, Wolong NNR and Heizhugou NNR. In Tangjiahe LAN, the YTM was directly associated with the hog badger, and the badger tended to occur in the range of the marten with a likelihood of 17–34% (table 6). They appeared in deciduous broadleaf forests and broadleaf–coniferous mixed forests with a slope of 6−30° at 1265–2397 m a.s.l. The marten was further associated with five other species and murids and the badger further with nine other species and murids (table 6). The association of the hog badger with the Malayan porcupine was mutually asymmetrical.

**Table 6.** Spatial associations of *M. flavigula* and *A. collaris* (refer to the caption in table 3).

network/NNR	species pair	the number of occurrence sites of Mustelids	the number of occurrence sites of related species	the number of occurrence sites	Phi coefficients (*rø*)	*L_A_* (upper）	*λ_A_* (lower）	*L_B_* (upper）	*λ_B_* (lower）
Heizhugou	yellow-throated marten (*M. flavigula*)–leopard cat (*P. bengalensis*)	10/28	10/28	7	0.53	—	—	—	—
Wolong LAN	yellow-throated marten (*M. flavigula*)–temminck's tragopan (*T. temminckii*)	21/60	17/60	11	0.39	—	—	—	—
Wolong LAN	yellow-throated marten (*M. flavigula*)–red panda (*Ailurus fulgens*)	21/60	18/60	14	0.59	0.48	0.23	0.39	0.06
Wolong LAN	yellow-throated marten (*M. flavigula*)–giant panda (*A. melanoleuca*)	21/60	19/60	12	0.4	—	—	—	—
Wolong LAN	yellow-throated marten (*M. flavigula*)–Asiatic black bear (*Ursus thibetanus*)	21/60	11/60	8	0.37	0.24	0.01	—	—
Wolong LAN	yellow-throated marten (*M. flavigula*)–Malayan porcupine (*Hystrix brachyura*)	21/60	20/60	13	0.44	—	—	—	—
Wolong LAN	yellow-throated marten (*M. flavigula*)–Sichuan snub-nosed monkey (*Rhinopithecus roxellana*)	21/60	23/60	15	0.5	0.33	0.03	0.39	0.13
Wolong LAN	yellow-throated marten (*M. flavigula*)–tufted deer (*Elaphodus cephalophus*)	21/60	39/60	21	0.54	—	—	—	—
Wolong LAN	yellow-throated marten (*M. flavigula*) sambar (*Rusa unicolor*)	21/60	45/60	21	0.42	—	—	—	—
Wolong LAN	yellow-throated marten (*M. flavigula*)–wild boar (*Sus scrofa*)	21/60	32/60	19	0.55	—	—	0.46	0.19
Wolong LAN	yellow-throated marten (*M. flavigula*)–forest musk deer (*Moschus berezovskii*)	21/60	13/60	11	0.55	0.43	0.01	—	—
Tangjiahe LAN	yellow-throated marten (*M. flavigula*)–leopard cat (*P. bengalensis*)	33/103	24/103	14	0.38	—	—	—	—
Tangjiahe LAN	yellow-throated marten (*M. flavigula*)–masked palm civet (*Paguma larvata*)	33/103	26/103	16	0.5	0.33	0.11	—	—
Tangjiahe LAN	yellow-throated marten (*M. flavigula*)–tufted deer (*E. cephalophus*	33/103	74/103	27	0.23	—	—	—	—
Tangjiahe LAN	yellow-throated marten (*M. flavigula*)–Malayan porcupine (*H. brachyura*)	33/103	45/103	22	0.28	—	—	0.21	0.04
Tangjiahe LAN	yellow-throated marten (*M. flavigula*)–David’s rock squirrel (*Sciurotamias davidianus*)	33/103	24/103	11	0.27	—	—	—	—
Tangjiahe LAN	yellow-throated marten (*M. flavigula*)–Muridae	33/103	12/103	10	0.46	0.3	0.15	—	—
Heizhugou	hog badger (*A. collaris*)–lady amherst’s pheasant (*Chrysolophus amherstiae*)	3/28	9/28	3	0.5	—	—	0.33	0.07
Heizhugou	hog badger (*A. collaris*)–masked palm civet (*P. larvata*)	3/28	9/28	3	0.5	—	—	0.33	0.07
Wolong HAN	hog badger (*A. collaris*)–snow partridge (*Lerwa lerwa*)	27/60	5/60	5	0.44	0.19	0.06	—	—
Wolong HAN	hog badger (*A. collaris*)–blood pheasant (*I. cruentus*)	27/60	16/60	13	0.33	0.37	0.18	—	—
Wolong HAN	hog badger (*A. collaris*)–Chinese monal (*L. lhuysii*)	27/60	15/60	11	0.33	0.26	0.06	—	—
Wolong HAN	hog badger (*A. collaris*)–red fox (*V. vulpes*)	27/60	12/60	10	0.38	0.3	0.12	—	—
Tangjiahe LAN	hog badger (*A. collaris*)–leopard cat (*P. bengalensis*)	40/103	24/103	15	0.27	0.18	0.002	—	—
Tangjiahe LAN	hog badger (*A. collaris*)–yellow-throated marten (*M. flavigula*)	40/103	33/103	23	0.42	0.34	0.17	—	—
Tangjiahe LAN	hog badger (*A. collaris*)–masked palm civet (*P. larvata*)	40/103	26/103	20	0.46	0.39	0.23	—	—
Tangjiahe LAN	hog badger (*A. collaris*)–Asiatic black bear (*U. thibetanus*)	40/103	56/103	27	0.25	—	—	—	—
Tangjiahe LAN	hog badger (*A. collaris*)–Malayan porcupine (*H. brachyura*)	40/103	45/103	26	0.37	0.27	0.05	0.33	0.15
Tangjiahe LAN	hog badger (*A. collaris*)–David’s rock squirrel (*S. davidianus*)	40/103	24/103	14	0.31	0.23	0.06	—	—
Tangjiahe LAN	hog badger (*A. collaris*)–Muridae	40/103	12/103	9	0.34	0.23	0.1	—	—
Tangjiahe LAN	hog badger (*A. collaris*)–rhesus macaque (*Macaca mulatta*)	40/103	6/103	5	0.26	0.14	0.04	—	—
Tangjiahe LAN	hog badger (*A. collaris*)–Sichuan snub-nosed monkey (*R. roxellana*)	40/103	57/103	30	0.3	—	—	—	—
Tangjiahe LAN	hog badger (A. collaris)–tufted deer (E.cephalophus)	40/103	74/103	33	0.26	—	—	—	—
Tangjiahe LAN	hog badger (*A. collaris*)–Chinese serow (*Capricornis milneedwardsii*)	40/103	29/103	18	0.31	0.23	0.05	—	—

In Wolong NNR, the two mustelids appeared in different communities and were not associated. The YTM was associated in Wolong LAN with ten non-mustelid species (table 6). Its associations with three non-mustelid species were uni-directionally asymmetrical, and the association with the Sichuan snub-nosed monkey was mutually asymmetrical. The hog badger was uni-directionally and asymmetrically associated with four non-mustelid species in Wolong HAN.

The two mustelids were not associated in Heizhugou. The YTM was associated only with leopard cats. The hog badger was associated with two non-mustelid species (table 6), and the associations were uni-directionally asymmetrical.

#### 3.3.5. The Siberian weasel

SWs were found in the four NNRs, but the species was interwoven only into Tangjiahe HAN where the weasel appeared in deciduous broadleaf forests, broadleaf–coniferous mixed forests, shrubs and meadows at 1805–3041 m a.s.l. The species was associated with four non-mustelid species (table 7). Its association with the Temminck’s tragopan connected the network with Tangjiahe LAN. In Liancheng, the weasel appeared in deciduous broadleaf forests and shrubs at 2022–3086 m a.s.l.; whereas in Wolong, it appeared in broadleaf–coniferous mixed forests and shrubs at 2782–3870 m a.s.l. where it stayed outside the networks.

**Table 7.** Spatial associations of *M. sibirica* (refer to the caption in table 3).

network/NNR	species pair	the number of occurrence sites of Mustelids	the number of occurrence sites of related species	the number of occurrence sites	Phi coefficients (*rø*)	*LA* (upper）	*λA* (lower）	*LB* (upper）	*λB* (lower）
Tangjiahe HAN	Siberian weasel (*M. sibirica*)–Temminck’s tragopan (*T. temminckii*)	9/103	27/103	5	0.41	—	—	—	—
Tangjiahe HAN	Siberian weasel (*M. sibirica*)–forest musk deer (*M. berezovskii*)	9/103	4/103	2	0.27	—	—	—	—
Tangjiahe HAN	Siberian weasel (*M. sibirica*)–chestnut-throated partridge (*T. obscurus*)	9/103	3/103	2	0.41	—	—	-—	—
Tangjiahe HAN	Siberian weasel (*M. sibirica*)–Gansu pika (*O. cansus*)	9/103	3/103	2	0.41	—	—	—	—

### 3.4. Spatial separation between mustelids

**Figure 6.**
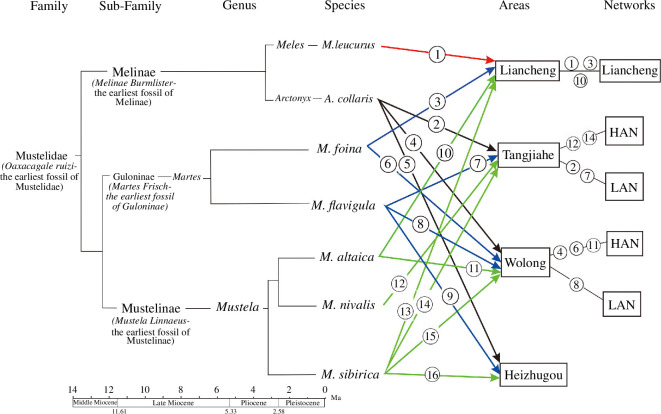
Divergences of the seven mustelids (Sato *et al.* [16]) and their spatial distribution. The circled numbers are guides to locate the spaces that the species occur.

Mustelid diversity was the lowest in Wolong LAN, with only one species (the YTM) occurring in the network (figure 6). There were more mustelid species in other networks, but direct associations between mustelids were found only in Wolong HAN and Tangjiahe LAN, i.e. the species pairs of the AW–SM and the YTM–hog badger. The two pairs accounted for <10% of the potential mustelid species pairs (*n* = 21), suggesting that mustelids tended to avoid spatial overlap in general.

Asian badgers from the subfamily Melinae appeared in the cold environment of the Liancheng network. According to infrared camera site data, the badger was found in the habitats of broadleaf–coniferous mixed forests, coniferous forests, shrubs and meadows at 1997–3041 m a.s.l. The hog badger from the same subfamily appeared in the warm environment of the three NNRs in Sichuan and was found in the same habitat types in Tangjiahe LAN and Wolong HAN, including broadleaf–coniferous mixed forests, coniferous forests, shrubs and meadow but at different altitudes which ranged 1264–2856 m a.s.l. in Tangjiahe LAN and 2743–4430 m a.s.l. in Wolong HAN (Table 8).

**Table 8.** Occurring frequencies (number of camera sites) of Melinae in different habitat dimensions. V = foot mountain valleys, L = lower parts of mountain bodies, M = middle parts of mountain bodies, U = upper parts of mountain bodies, R = mountain ridges, NR = not recorded, E = eastern, S = southern, W = western, *n* = northern, NE = northeastern, SE = southeastern, NW = northwestern, SW = southwestern, CBM = coniferous and broadleaf mixed forests, CF = coniferous forests, DB = deciduous broadleaf forests, EBM = evergreen and deciduous broadleaved mixed forest, EGB = evergreen broadleaf forests, BS = bushes, MD = meadow, SC = screes.

mustelids (networks)	the number of sites of Species	altitude range (m a.s.l.)	mountain levels	mountain aspects	slope	vegetation types	vegetation height	understory bush height	understory bush coverage (%)	meadow coverage (%)
Asian badger (*M. leucurus*) (Liancheng)	72/128	1997–3086	NR = 72	NR = 72	NR: 72	DB: 15; CBM: 11; CF: 31; MD:1; BS:14	NR: 72	NR: 72	NR: 72	NR: 72
hog badger (*A. collaris*) (Tangjiahe LAN)	44/103	1264–2856	L = 4, M = 13, U = 10, R = 7, NR = 10	E = 4, S = 12, W = 4, *n* = 8, NE = 3, SW = 1, NR = 12	0–5°: 1; 6–20°: 17; 21–30°:15; NR: 11	DB: 19; CBM: 15; NR: 10	5–9 m: 1; 10–19 m: 32; NR: 11	1–3 m: 31; 3–5 m: 3; NR: 10	0–24: 7; 25–49: 20; 50–74: 7; NR: 10	0–24: 7; 25–49: 22; 50–74: 5; NR: 10
hog badger (*A. collaris*) (Wolong HAN)	27/60	2743–4430	V = 9, L = 5, M = 4, U = 5, R = 4	W = 4, *n* = 1, NE = 5, SE = 1, NW = 9, SW = 6, NR = 1	0–5°: 8; 6–20°: 7; 21–30°:7; 31–40°: 5	DB: 1; CBM: 12; CF: 2; BS: 7; MD: 1; SC:4	0 m: 5; 1–4 m: 7; 5–9 m: 1; 10–19 m: 8; 20–29 m: 6	0 m: 5; 0–1 m: 2; 1–3 m: 3; 3–5 m: 17	0: 5; 0–24: 13; 25–49: 7; 50–74: 2	0: 4; 0–24: 14; 25–49: 5; 50–74: 3; 75–100: 1
hog badger (*A. collaris*) (Heizhugou NNR)	3/28	2260–2467	M = 1, U = 1, R = 1	NW = 1, NR = 2	0–5°: 3	EGB: 1; CBM: 1; EBM: 1	NR: 3	0–1 m: 2; 1–3 m: 1	NR: 3	0–24: 2; 75–100: 1

The SM and the YTM from the genus *Martes* (subfamily Guloninae) were found in different climate zones; the SM occurred in the cold weather in Liancheng and Wolong HAN and the YTM in the warm weather in Tangjiahe LAN and Wolong LAN. The SM was found in the deciduous broadleaf forests, broadleaf–coniferous mixed forests and coniferous forests at 1997–3041 m a.s.l. in Liancheng and the shrubs and meadows at 4052–4338 m a.s.l. in Wolong HAN. The YTM was found in deciduous broadleaf forests and broadleaf–coniferous mixed forests at 1220–2397 m a.s.l. in Tangjiahe LAN, deciduous broadleaf forests, broadleaf–coniferous mixed forests and coniferous forests at 1979–3361 m a.s.l. in Wolong LAN. This showed that the two *Martes* species occurred in similar habitats, but the habitat range of the SM was wider, reaching to the colder alpine shrubs and meadows in Wolong HAN (table 9).

**Table 9.** Occurring frequencies (number of camera sites) of *Martes* in different habitat dimensions (refer to the caption in table 8).

mustelids (networks/area)	the number of sites of Species	altitude range (m a.s.l.)	Mountain levels	mountain aspects	slope	vegetation types	vegetation height	understory bush height	understory bush coverage (%)	grass coverage (%)
stone marten (*M. foina*) (Liancheng)	29/128	2011–3086	NR = 29	NR = 29	NR: 29	DB: 7; CBM: 3; CF: 18; MD: 1	NR: 29	NR: 29	NR: 29	NR: 29
stone marten (*M. foina*) (Wolong HAN)	5/60	4052–4338	V = 2, L = 1, M = 2	W = 2, NE = 1, NW = 2	6–20°: 1; 21–30°: 2; 31–40°: 2	BS: 2; MD: 1; SC: 2	0 m: 3; 0–1 m: 2	0 m: 3; 0–1 m: 2	0: 3; 25–49: 1; 50–74: 1	0: 2; 0–24: 1; 25–49: 2
yellow-throated marten (*M. flavigula*) (Tangjiahe LAN)	33/103	1220–2397	V = 1, L = 1, M = 9, U =11, R = 3, NR = 8	E = 1, S = 11, W = 4, *n* = 7, NE = 1, NR = 9	6–20°: 15; 21–30°: 9; NR: 9	DB: 14; CBM: 11; NR: 8	5–9 m: 1; 10–19 m: 23; NR: 9	0–1 m: 1; 1–3 m: 22; 3–5 m: 2; NR: 8	0–24: 5; 25–49: 15; 50–74: 5; NR: 8	0–24: 6; 25–49: 15; 50–74: 4; NR: 8
yellow-throated marten (*M. flavigula*) (Wolong LAN)	21/60	1979–3361	V = 7, L = 4, M = 4, U = 1, R = 5	S = 1, *n* = 1, NE = 4, SE = 4, NW = 8, SW = 2, NR = 1	6–20°: 5; 21–30°: 7; 31–40°: 1	DB: 3; CBM: 14; CF: 3BS: 1	1–4 m:1; 5–9 m: 2; 10–19 m: 14; 20–29: 4	1–3 m: 4; 3–5 m: 17	0–24: 12; 25–49: 8; 50–74: 1	0–24: 11; 25–49: 3; 50–74: 7
yellow-throated marten (*M. flavigula*) (Heizhugou NNR)	10/28	2260–3018	V = 4, L = 2, M = 2, U = 1, R = 1	E = 2, NE = 3, SE = 2, NW = 1, NR = 2	0–5°: 8; 6–20°: 1; 21–30°:1	EGB: 1; CBM: 6; CF: 2; EBM: 1	NR: 10	0–1 m: 2; 1–3 m: 4; 3–5 m: 4	NR: 10	0–24: 3; 25–49: 1; 50–74: 3; 75–100: 3

The AW and the LW, two species from the genus *Mustela*, were found in the cold environments of Liancheng (*M. altaica*), Wolong HAN (*M. altaica*) and Tangjiahe HAN (*M. nivalis*). The AW appeared in deciduous broadleaf forests, broadleaf–coniferous mixed forests, coniferous forests, shrubs and meadows at 2022–3026 m a.s.l. in Liancheng. It occurred in similar habitats to those of the SM in Wolong HAN but at different altitudes ranging from 4303 to 4338 m a.s.l. in Wolong HAN. The LW was found in the shrubs and meadows habitats at 3041 m a.s.l. in Tangjiahe HAN (table 10).

**Table 10.** Occurring frequencies (number of camera sites) of *M. altaica* and *M. nivalis* in different habitat dimensions (refer the caption in table 8).

mustelids (networks)	the number of sites of Species	altitude range (m a.s.l.)	mountain levels	mountain aspects	slope	vegetation types	vegetation height	understory bush height	understory bush coverage (%)	grass coverage (%)
Altai weasel (*M. altaica*) (Liancheng)	10/128	2022–3026	NR = 10	NR = 10	NR: 10	DB: 2; CBM: 4; CF: 3; BS: 1	NR: 10	NR: 10	NR: 10	NR: 10
Altai weasel (*M. altaica*) (Wolong HAN)	2/60	4303–4338	L = 1, M = 1	W = 1, NW = 1	21–30°:1; 31–40°: 1	SC: 2	0 m: 2	0 m: 2	0: 2	0: 2
least weasel (*M. nivalis*) (Tangjiahe HAN)	1/103	3041	NR = 1	NR = 1	NR: 1	NR: 1	NR: 1	NR: 1	NR: 1	NR: 1

The SW from the genus *Mustela* was found in a wider climate zone covering both warm and cold areas but stayed outside the networks except for the population in Tangjiahe where it was interwoven in the HAN. The weasel appeared in deciduous broadleaf forests, broadleaf–coniferous mixed forests, shrubs and meadows at 1805–3041 m a.s.l. in Tangjiahe HAN, perhaps indicating that the species preferred a cold environment (table 11).

**Table 11.** Occurring frequencies (number of camera sites) of *M. sibirica* in different habitat dimensions (refer to the caption in table 8).

mustelids (networks/area)	the number of sites of Species	altitude range (m a.s.l.)	mountain levels	mountain aspects	slope	vegetation types	vegetation height	understory bush height	understory bush coverage (%)	grass coverage (%)
Siberian weasel (*M. sibirica*) (Liancheng NNR)	6/128	2022–3086	NR = 6	NR = 6	NR: 6	DB: 5; CBM: 1	NR: 6	NR: 6	NR: 6	NR: 6
Siberian weasel (*M. sibirica*) (Wolong NNR)	2/60	2752–3870	V = 1, L = 1	W = 1, NR = 1	0–5°: 1; 21–30°: 1	CBM: 1; BS: 1	3–5 m: 1; 10–19 m: 1	3–5 m: 2	0–24: 1; 50–74: 1	0–24: 2
Siberian weasel (*M. sibirica*) (Tangjiahe HAN)	7/103	1805–3041	M = 2, U = 3, NR = 2	E = 2, S = 1, W = 1, *n* = 1, NR = 2	6–20°: 2; 21–30°: 3; NR: 2	DB: 2; CBM: 3; NR: 2	10–19 m: 4; 20–29 m: 1; NR: 2	1–3 m: 4; 3–5 m: 1; NR: 2	25–49: 4; 50–74: 1; NR: 2	25–49: 5; NR: 2
Siberian weasel (*M. sibirica*) (Heizhugou NNR)	9/28	2378–2745	V = 3, L = 3, M = 2, U = 1	E = 2, NE = 1, SE = 2, NW = 2, SW = 2	0–5°: 5; 6–20°: 1; 21–30°: 1	CBM: 6; CF: 2; SC: 1	NR: 9	0 m: 1; 1–3 m: 5; 3–5 m: 3	NR: 9	0:1; 0–24: 1; 25–49: 3; 50–74: 2; 75–100: 2

## 4. Discussion

A total of seven mustelids were found in our data, including *Meles leucurus*, *Arctonyx collaris*, *Martes foina*, *Martes flavigula*, *Mustela altaica*, *Mustela nivalis* and *Mustela sibirica*. According to Sato *et al*. [17], *Meles* and *Arctonyx* belong to the subfamily Melinae*, Martes* belongs to Guloninae and *Mustela* belongs to Mustelinae*.* Accordingly, the species pairs of *M. leucurus–M. foina* and *M. altaica*, *A. collaris–M. flavigula*, *A. collaris–M. foina* and *M. altaica*, *M. foina–M. altaica* and *M. nivalis–M. foina* are pairs between subfamily, *M. leucurus–A. collaris* is a pair between genera, and *M. foina–M. flavigula and M. nivalis–M. sibirica* are pairs within genus*.*

### 4.1. Spatial distribution between subfamily

#### 4.1.1. *Meles leucurus*, *Martes foina* and *Mustela altaica*

Our results indicate that the Asian badger (*M. leucurus*) from the Melinae, the SM (*M. foina*) from the Guloninae and the AW (*M. altaica*) from the Mustelinae were present in the Liancheng network (figure 2). However, they lived in different habitat types and avoided spatial associations.

The three mustelids all prefer shrubs. They are all burrow dwellers and feed on small mammals, amphibians, reptiles and birds [33,38–40], indicating similar ecological habits that favour colder communities. However, the Asian badger has a large body weight (3.5–9 kg), short limbs and a stubby tail [41]. The body weight of the AW ranges from 80 to 280 g. In comparison, the SM weighs between 800 and 1600 g [33,41], making it significantly heavier than the AW but lighter than the Asian badger. The Asian badger is typically found in densely forested areas. In addition to shrubs, it also inhabits broadleaf–coniferous mixed forests and coniferous forests [33]. The AW inhabits open habitats like meadows, screes, dry grasslands and river valleys with reed shrubs [41,42]. The SM shares shrub meadows and forests with the badger but is also found in riverbanks, orchards, pastures and screes [43]. They use crevices in rocks, rock piles or holes abandoned by other animals [41]. The badger is a nocturnal and social animal that inhabits cold climates [33] and often uses burrows abandoned by marmots. In addition to animal foods, it also feeds on fruits, leaves and insects [38,39]. The weasel is a cave dweller, but it does not build fixed nests [44]. The diet of the weasel is more limited than that of the badger. It primarily preys on rodents, pikas and hatchlings [39,40]. The marten feeds on fruits, arthropods, pikas, rodents and birds [45,46]. These differences in ecological habits allowed the mustelids to inhabit various habitat types within the same community (i.e. habitat niche separation).

#### 4.1.2. *Arctonyx collaris* and *Martes flavigula*

Our results showed that the hog badger (*A. collaris*) from the Melinae and the YTM (*M. flavigula*) from the Guloninae coexisted in Tangjiahe LAN, and their association was uni-directionally asymmetric (figure 3), in which the badger tended to occur in the marten’s range with a likelihood of 17–34%.

The hog badger inhabits broadleaf–coniferous mixed forests with dense canopies and steep slopes, characterized by high shrub density and low tree coverage. The hog badger uses stone crevices and natural caves as nesting sites [47]. The YTM inhabits the same type of vegetation but prefers areas rich with fallen logs [48]. Both species are diurnal; living in tree holes or caves; and feeding on fruits, birds and rodents. In addition, the badger has an omnivorous diet, consuming roots, stems, invertebrates, amphibians and reptiles [49]. In contrast, the marten’s diet includes insects and small- and medium-sized mammals [48,50]. The results of this study also showed that they were jointly associated with five animal species, including the Malayan porcupine (*Hystrix brachyura*), tufted deer (*Elaphodus cephalophus*), leopard cat (*Prionailurus bengalensis*), David’s rock squirrel (*Sciurotamias davidianus*) and masked palm civet (*Paguma larvata*), and shared a similar habitat type (tables 8–9) in Tangjiahe LAN. It may be the similarities in diet and habitat type that allow the two species to coexist in the community environment. The Lambda statistic test may indicate a commensal interaction underlying the association between them [27]. The two mustelids share some plants in their diets, such as *Actinidia chinensis*, *Clematoclethra scandens*, *Sorbus hemsleyi*, *Diospyros lotus*, *Hovenia dulcis* and *Celtis biondii* [50,51]. The marten is a fruit eater that disperses seeds after consuming them [52,53], thereby facilitating the germination of plants [52]. This behaviour may increase the frequency of plants within its range. This may make the marten’s habitats more attractive to the badger, as the badger find food more easily there.

#### 4.1.3. *Arctonyx collaris*, *Mustela altaica* and *Martes foina*

Our findings revealed that the hog badger (*A. collaris*) from the Melinae, the AW (*M. altaica*) from the Mustelinae and the SM (*M. foina*) from the Guloninae were present in Wolong HAN. The marten tended to be found in the same area as the weasel with a probability ranging from 4 to 40%, while the hog badger avoided spatial associations with the weasel and the marten (figure 4).

Our data from camera sites showed that the hog badger was present in the areas at 2473–4430 m a.s.l. in Wolong HAN, whereas the AW and the SM were found above 4000 m a.s.l. (tables 8–10). The badger weighs 9.7–12.5 kg [41], whereas the weasel and the marten weigh only 80–280 g and 800–1600 g, respectively [33,41]. The badger prefers broadleaf–coniferous mixed forests with high canopy density, whereas the weasel prefers shrub meadows and screes. Although the badger and marten inhabit similar habitats, such as broadleaf–coniferous mixed forests and forest margins, the marten shows a preference for shrub meadows, screes, river banks, orchards and pastures [41,43,54]. All of these findings indicate that the three mustelids have different preferred habitats, which include forested areas, forest edges and open habitats. These differences place them in separate categories.

The presence of badgers in Tangjiahe LAN and their absence in Tangjiahe HAN indicates that badgers prefer warm habitats. However, it occurred in Wolong HAN. According to our analysis [55], cattle grazing at low altitudes in Wolong led to damage to the badgers’ dwelling caves, while such grazing did not occur in Tangjiahe. Therefore, it was the cattle grazing that forced the badger to adapt to survive at high altitudes in Wolong. This also indicates that the badger should have evolved some strategies to survive in cold environments, even though it prefers warm environments.

#### 4.1.4. *Mustela altaica* and *Martes foina*

The AW (*M. altaica*) and the SM (*M. foina*), two mustelids co-occurring in Liancheng network and Wolong HAN, represent the differentiation between the subfamilies Mustelinae and Guloninae within the Mustelidae in this study. Mustelinae and Guloninae may have diverged in the middle Miocene (figure 6). According to Fossilworks [56], the earliest fossil of *Mustela* from the Mustelinae was discovered in the early Miocene (20.4−16 mabp) deposits in Jiangsu, China. Subsequent fossils were discovered in the middle and late Miocene (16–5.3 mabp) deposits in Southern Europe and North America, suggesting that the genus may have originated in eastern China and spread westward to the area of this study. The earliest fossil of *Martes* from the Guloninae was found in early Miocene Europe and western Asia (23–16.9 mabp), with younger fossils discovered in the middle and late Miocene (16–8.7 mabp) in East Asia, Central Asia, Southeast Asia and North America. This suggests that the genus may have originated in Europe and spread eastward to the study area. The SM diverged from the *Martes* genus in Europe during the late Pliocene (2.67 mabp) [57] and migrated eastward to interact with the AW, which diverged in East Asia during the late Pliocene (2.8 mabp) [20]. Both species have adapted to glacial climates and have developed cold tolerance.

The AW and the SM are both small predators with similar feeding habits and habitat types [39–46,54]. However, the marten is larger in body size [32,41], dwells in a wider range of habitats, has a more varied diet and is distributed over a larger geographic area. In our results, they may coexist in the Liancheng network and Wolong HAN owing to their similarities. In the Liancheng network, the two species were connected by chestnut-throated partridges. In Wolong HAN, they were directly associated with a high coefficient (*r_ø_* = 0.62), which may be related to predatory interactions. Our Lambda statistic test results showed a uni-directional asymmetric association, indicating that the marten tended to occur within the range of the weasel. Donadio & Buskirk [58] suggest that interspecific killing to eliminate competitors is more likely to occur if the two species in question are moderately different in body size (2–5.4 times), have adapted to preying on vertebrates, overlap in diets and are from the same family. The body size difference between the marten (800–1600 g) and the weasel (80–280 g) is 5.7–10 times [33,41], which is considered a moderate difference. Both species prey on rodents and birds [33], have overlapping diets and belong to the same family Mustelidae, indicating that all conditions for interspecific killing have been met between the two species [59]. The marten’s teeth are short, conical and cultrate in shape. It also possesses chimeric cleft teeth [60], indicating its more predatory nature and ability to tear prey. Thus, the SM may kill the weasel to eliminate competition, which could explain why the marten is found in the same range as the weasel. The interspecific killing may also deter the two mustelids from directly associating in the Liancheng network. In general, killing occurred in only one pair of the 21 potential species pairs, accounting for 4.76%. This behaviour was not common in our study area, possibly owing to the mountain habitats (interspecific killing is common in savanna habitats [58]). Thus, it may not play a significant role in reducing the biodiversity of the family, although it may decrease the biodiversity within the family.

#### 4.1.5. *Mustela nivalis* and *Martes foina*

In our results, the LW (*M. nivalis*) from the Mustelinae and the SM (*M. foina*) from the Guloninae both showed a preference for alpine shrubs and meadows (tables 9–10), indicating their similarity in ecological requirements. The weasel was found in Tangjiahe HAN, whereas the marten was found in Wolong HAN, indicating spatial separation among mustelids with similar ecological characteristics to avoid competition.

### 4.2. Spatial distribution between genera: *Meles leucurus* and *Arctonyx collaris*

The Asian badger (*M. leucurus*) was found in the Liancheng network in Gansu, whereas the hog badger (*A. collaris*) was found in the three NNRs in Sichuan province. The distinction represents the differentiation between the genera *Meles* and *Arctonyx* from the subfamily Melinae in this research. The oldest *Meles* fossil was found in a late Miocene deposit in Greece, whereas the younger fossils have been discovered in Pliocene and Pleistocene deposits across Eastern Europe, Central and East Asia, and China [19]. This suggests that the genus originated in the Mediterranean and spread eastward. The Asian badger diverged from *Meles meles* in the late Pliocene (2.92 mabp) [19]. The oldest *Arctonyx* fossil was found in a late Pliocene (2.8 mabp) deposit in Hunan province, South China. Younger fossils have been discovered in middle Pleistocene deposits in Guangdong and Anhui provinces, as well as in Vietnam [56], suggesting that the genus originated in South China. During the eastward dispersal from the Mediterranean, the *Meles* encountered glacial climates. The locally originated *Arctonyx* experienced monsoon climates while dispersing westward in the area under study. Accordingly, *Meles* has been adapted to cold habitats, enabling them to inhabit communities in North China, such as the Liancheng network in this research. In contrast, *Arctonyx* has been adapted to habitats with highly variable temperatures but prefers warm habitats, enabling it to inhabit warm communities like the Tangjiahe LAN, as well as cold communities such as the Wolong HAN. The hog badger’s flexibility is still reflected in its extant distribution in diverse habitats but mainly in warm areas [61]. It may be because of the flexibility that enables it to avoid the Asian badger. The hog badger occurred in Wolong HAN because of avoiding human disturbance [55].

### 4.3. Spatial distribution within genus

#### 4.3.1. *Martes foina* and *Martes flavigula*

The SM (*M. foina*) and the YTM (*M. flavigula*) belong to the genus Martes. The YTM diverged from the genus in the late Miocene (5.48 mabp) [20,57]. The species is currently distributed in eastern Russia, Southeast Asia, southern China, the Himalayas and India [62]. This distribution indicates that the species has adapted to warm communities, such as Tangjiahe LAN, Wolong LAN and Heizhugou NNR as observed in this study. The SM diverged from its ancestral species in the late Pliocene (2.67 mabp) [57], with the earliest fossil found in Pleistocene western Asia [56]. It is currently distributed in Europe, Central Asia and western China [54]. During the dispersal of the SM, it may have encountered the Pleistocene glacial climates and evolved the ability to survive in the cold communities of eastern Asia, including the Liancheng network and Wolong HAN mentioned in this study. However, the habitat preferences of the two species suggest that the SM prefers warmer environments, and its presence in the cold environment of Wolong HAN may be owing to competition with the YTM. Data from Liancheng network and Wolong HAN (table 9) showed that the habitats of the SM range from deciduous broadleaf forests and broadleaf–coniferous mixed forests to coniferous forests and alpine shrubs and meadows. In Liancheng, where the YTM was not appearing, the SM occurred in the forests. In contrast, in Wolong, where the YTM appeared, the SM occurred in the alpine shrubs and meadows (table 9). We believe that the SM avoids competition by relocating to cold communities where the YTM cannot survive owing to their high degree of ecological requirements similarity (competitive exclusion [63]).

The SM was also found in the same area as *M. leucurus* and *M. altaica* in the Liancheng network, but there were no direct spatial associations among them. The YTM was asymmetrically associated with *A. collaris* (subfamily Melinae) in Tangjiahe LAN. The associations of these species pairs have been discussed earlier.

#### 4.3.2. *Mustela altaica*, *Mustela nivalis* and *Mustela sibirica*

The AW (*M. altaica*), LW (*M. nivalis*) and SW (*M. sibirica*) belong to the genus *Mustela*. The AW and the LW, the two smallest members of the Mustelid family, are closely related and diverged in the late Pliocene (2.8 mabp) [20]. The oldest AW fossil was found in Anhui (2.588 mabp), whereas the oldest LW fossils were discovered in western Asia (Georgia) and North America (2.6 mabp) [56]. These findings suggest that the LW may have originated in western Asia and dispersed eastward to the area of this study, whereas the AW may have originated in eastern China and dispersed westward to the area. During their dispersal from western Asia, the LW experienced the Pleistocene glacial climates, which may have led to the evolution of adaptations for cold environments. Anhui was in a glacial climate during the Pleistocene [64], suggesting that the AW may have originated and evolved in cold habitats. This evolutionary history may be the basis for the occurrence of the two species in cold communities, such as Liancheng network, Tangjiahe HAN and Wolong HAN as observed in this study.

The AW occurred in Lianchang network and Wolong HAN. The LW occurred in Tangjiahe HAN. The results are shown in table 10. Both species exhibited a preference for alpine shrubs and meadows at high altitudes but in different geographical regions, indicating a distribution pattern of vicariance [65].

The SW was present in all of the NNRs in this study, but it was only associated with other species [the forest musk deer (*Moschus berezovskii*), Temminck’s tragopan (*Tragopan temminckii*), chestnut-throated partridge (*Tetraophasis obscurus*) and Gansu pika (*Ochotona cansus*)] in Tangjiahe HAN and remained outside all other species networks. It was not associated with any mustelids. Law *et al.* [19] suggest that the SW diverged from the group of *M. nivalis–M. altaica* [20] in the early Pleistocene (1.9 mabp), but no convincing fossil records are available yet to indicate its place of origin. Its current distribution spans altitudes ranging from 1500 to 5000 m a.s.l. and includes various habitat types such as forests, plains, shrubs and village farmlands. It preys on murids, wild rabbits, amphibians and insects and also feeds on seeds [33]. These dimensions indicate its adaptation to diverse habitats. However, it is not yet known why the SW was associated with other species in Tangjiahe HAN but remained outside of most networks. Therefore, further studies are needed to explore the reasons.

### 4.4. Mustelids in the four national nature reserves

The four NNRs have experienced different evolutionary history. The weather in northwest China where Liancheng NNR is located started to become dry and cold in Pliocene [66]. With the evolution from subtropical wetland vegetation and humid forests in Miocene to dry- and cold-tolerant grassland and desert grassland in Pliocene, the warm and humid loving faunal components (such as *Platybelodon*, *Pliopithecus* and *Limnopithecus*) had been replaced by the cold- and dry-tolerant components (such as *Struthio linxiaensis*, *Acinonyx* and *Coelodonta antiquitatis*) [1]. Our data recognized only one species network in Liancheng NNR. This may indicate that the altitudinal difference (1746 m) was not great enough to support significant divergence of biological community; thus, all species stayed in the same community.

The warm and humid air currents from the southwest enhanced the humidity in southwest China where Tangjiahe, Wolong and Heizhugou NNRs are located. This resulted in mountain glaciers at high altitudes since the Pliocene [31,66]. Thus, the environment differentiation occurred in humidity between Liancheng and the southern NNRs, in temperature between high and low altitudes in the southern NNRs and in humidity and temperature between Liancheng and the low altitudes in the southern NNRs.

Such an environment differentiation pattern may be the ecological basis for the spatial distribution of mustelids. Liancheng NNR, Tangjiahe HAN and Wolong HAN would house cold-tolerant species, whereas Tangjiahe LAN and Wolong LAN would house warm loving species. Our data showed that all the subfamilies that occur in the study area, i.e. Melinae, Guloninae and Mustelinae, occurred in all the NNRs, meaning that the differentiation in distribution does not occur at subfamily level; instead, it occurs at lower taxa. The Asian badger (*M. leucurus*), SM (*M. foina*) and LW (*M. nivalis*) are cold-tolerant species [31] and would enter Liancheng network, Tangjiahe HAN and Wolong HAN, and hog badger (*A. collaris*) and YTM (*M. flavigula*) love warm environments [31] and thus would enter Tangjiahe LAN and Wolong LAN, which are supported by our results. The SW (*M. sibirica*) occurred in all NNRs, but stayed outside species networks in Heizhugou and Wolong NNRs. However, it was interwoven in Tangjiahe HAN. So, the species may be adapted to cold and humid communities where the terrain is relatively flat.

Comparisons on the number of mustelid species, the number of species associations and the number of asymmetrical associations do not show changes from one network to the other owing to the environmental characteristics of the networks. The disappearance of network from Heizhugou NNR may be attributed to severe deforestation before the area became protected in the 1990s, and the current appearance shown in figure 5 may be the state of community succession [22,25]. Well protection of the hog badger may facilitate the succession to connect the species into networks because Lady amherst’s pheasant (*Chrysolophus amherstiae*) and masked palm civet (*P. larvata*) tended to attend in the range of the badger. Our analysis on uni-directionally asymmetrical associations of primates with other species suggests that a species tends to attend in another species’ range because it benefits from the latter species [25]. Thus, Lady amherst’s pheasant (*C. amherstiae*) and masked palm civet (*P. larvata*) may benefit from the badger by attending in the badger’s range. If a HAN is recognized in Heizhugou NNR, the SW may be interwoven into the community because the community will be located in cold, humid and flat terrain. So, the conservation of mustelids in Heizhugou will not only benefit them *per se* but also benefit other species.

## 5. Conclusion

It is concluded from the above discussions that differentiation in spatial distribution occurs at the levels of genus and species. The Asian badger (*Meles leucurus*) and the SM (*Martes foina*) diverge at genus level and occur in different climate zones and at different altitudes. Because of their similar ecological requirements, species from the same genus avoid spatial overlap either by vicariance [e.g. the AW (*Mustela Altaica*) and the LW (*Mustela nivalis*)] or by competitive exclusion [e.g. the YTM (*Martes flavigula*) and the SM]. Other mustelids in the same communities avoid spatial overlap by inhabiting different habitat types. Thus, mustelids rarely meet each other in natural communities. The hog badger (*Arctonyx collaris*) was directly associated with the YTM in Tangjiahe LAN and tended to occur in the marten’s range perhaps because the badger benefits from the seed dispersing by the marten. This interaction will not negatively impact the species diversity of mustelids. The SM was directly associated with the AW in Wolong HAN and tended to occur in the weasel’s range perhaps because the marten kills the weasel to eliminate competition. However, this interaction (*n* = 1) accounts for only a small proportion of interspecific interactions between mustelids (*n* = 21) and may not significantly impact the biodiversity of the family. Therefore, our research findings support the predictions we propose in this article.

Comparisons show that the species number of mustelids and the number of their associations did not change across the NNRs owing to the environment characteristics of the NNRs. However, the adaptation of the mustelids to temperature may influence them when they entered biological communities. Competition sometimes may also play a role in this respect. The asymmetrical associations of the mustelids with non-mustelids may facilitate the development of the non-mustelids. Thus, protection of the mustelids may benefit the conservation of overall biodiversity in Heizhugou, an NNR that has experienced severe deforestation.

## Data Availability

Data of the occurrence of different species at each camera site and Species List of Terrestrial Animal in four national nature reserves: Dryad [67].

## References

[1] Li Z , Dong X . 2023 Chapter 5: historical changes of some important animal distribution areas. In Zoogeography (eds Q Shi , D Bai ), pp. 169–177. Beijing: Science Press.

[2] Marin J *et al* . 2018 Evolutionary time drives global tetrapod diversity. Proc. R. Soc. B **285** , 20172378. (10.1098/rspb.2017.2378) PMC5829197 29436494

[3] Allen AP , Gillooly JF , Brown JH . 2007 Recasting the species-energy hypothesis: the different roles of kinetic and potential energy in regulating biodiversity. In Scaling biodiversity (eds D Storch , PA Marquet , JH Brown ), pp. 283–299. Cambridge, UK: Cambridge University Press. (10.1017/CBO9780511814938)

[4] Evans KL , Warren PH , Gaston KJ . 2005 Species energy relationships at the macroecological scale: a review of the mechanisms. Biol. Rev. Camb. Philos. Soc. **80** , 1–25. (10.1017/s1464793104006517) 15727036

[5] Hurlbert AH , Jetz W . 2010 More than ‘more individuals’: the nonequivalence of area and energy in the scaling of species richness. Am. Nat. **176** , E50–65. (10.1086/650723) 20184428

[6] Fløjgaard C , Normand S , Skov F , Svenning JC . 2011 Deconstructing the mammal species richness pattern in Europe-towards an understanding of the relative importance of climate, biogeographic history, habitat heterogeneity and humans. Glob. Ecol. Biogeogr. **20** , 218–230. (10.1111/j.1466-8238.2010.00604.x)

[7] Baselga A , Lobo JM , Svenning J , Aragón P , Araújo MB . 2012 Dispersal ability modulates the strength of the latitudinal richness gradient in European beetles. Glob. Ecol. Biogeogr. **21** , 1106–1113. (10.1111/j.1466-8238.2011.00753.x)

[8] Nosil P . 2012 Ecological Speciation. Oxford: Oxford University Press. (10.1093/acprof:osobl/9780199587100.001.0001)

[9] Gulick JT . 1888 Divergent evolution through cumulative segregation. J. Linnean Soc. Lond. Zool. **20** , 189–274. (10.1111/j.1096-3642.1888.tb01445.x)

[10] Hvala JA , Wood TE . 2012 Speciation: introduction. Evol. & Divers. Life. 1–10. (10.1002/9780470015902.a0001709.pub3)

[11] Futuyma DJ . 2017 Chapter 8: Formation of species. In Evolution (ed. S Ge ), pp. 466–472. Sunderland: Sinauer Associates Inc.

[12] Pocheville A *et al* . 2015 The ecological niche: history and recent controversies. In Handbook of evolutionary thinking in the sciences (eds T Heams , P Huneman , G Lecointre ), pp. 547–586. Dordrecht: Springer.

[13] Hardin G . 1960 The competitive exclusion principle. Science **131** , 1292–1297. (10.1126/science.131.3409.1292) 14399717

[14] Wund M . 2005 “Mustelidae“. Animal Diversity Web. See https://animaldiversity.org/accounts/Mustelidae (accessed 30 December 2020).

[15] Vaughan T , Ryan J , Czaplewski N . 2017 Chapter 18: general order - Carnivora. In Mammalogy, pp. 188–189. Beijing: Science Press.

[16] Koepfli KP , Deere KA , Slater GJ , Begg C , Begg K , Grassman L , Lucherini M , Veron G , Wayne RK . 2008 Multigene phylogeny of the Mustelidae: resolving relationships, tempo and biogeographic history of a mammalian adaptive radiation. BMC Biol. **6** , 1–22. (10.1186/1741-7007-6-10) 18275614 PMC2276185

[17] Sato JJ , Wolsan M , Prevosti FJ , D’Elía G , Begg C , Begg K , Hosoda T , Campbell KL , Suzuki H . 2012 Evolutionary and biogeographic history of weasel-like carnivorans (Musteloidea). Mol. Phylogenet. Evol. **63** , 745–757. (10.1016/j.ympev.2012.02.025) 22410652

[18] Marmi J , López‐Giráldez JF , Domingo‐Roura X . 2004 Phylogeny, evolutionary history and taxonomy of the Mustelidae based on sequences of the cytochrome b gene and a complex repetitive flanking region. Zool. Scr. **33** , 481–499. (10.1111/j.0300-3256.2004.00165.x)

[19] Law CJ , Slater GJ , Mehta RS . 2018 Lineage diversity and size disparity in mustelidea: testing patterns of adaptive radiation using molecular and fossil-based methods. Syst. Biol. **67** , 127–144. (10.1093/sysbio/syx047) 28472434

[20] Hosoda T , Suzuki H , Harada M , Tsuchiya K , Han SH , Zhang Y , Kryukov AP , Lin LK . 2000 Evolutionary trends of the mitochondrial lineage differentiation in species of genera Martes and Mustela. Genes Genet. Syst. **75** , 259–267. (10.1266/ggs.75.259) 11245219

[21] Ricklefs R . 2008 Chapter 14: Species interactions. In The economy of nature, pp. 287–302, Sixth Edition. New York: WH Freeman and Company.

[22] Liu Z , Shen L , Li Z , Zhou H , Li Q , Wang X . 2023 Species associations and conservation of giant pandas. Glob. Ecol. Conserv. **43** , e02428. (10.1016/j.gecco.2023.e02428)

[23] Zhou H , Jiang N , Li J , Yang H , Wu Y , Shi X , Li Z . 2021 Study on the terrestrial animal community that Panthera uncia lived in Wolong national nature reserve, Sichuan province, China. Chin. J. Wildl. **42** , 645–653. (10.19711/j.cnki.issn2310-1490.20210616.001)

[24] Su T , Cui G , Man Z , Li W , Huang Z , Chen J , Zhao M . 2023 Interspecific association of Sika deer in terrestrial animal communities of Liancheng national nature reserve, China. Integr. Zool. **18** , 688–703. (10.1111/1749-4877.12700) 36549005

[25] Li Q , Li Z , Liu Z . 2024 Spatial Association networks reveal the biological communities of the Tibetan Macaque (Macaca thibetana) in Sichuan, China. Int. J. Primatol. (10.1007/s10764-024-00417-7)

[26] Wang X , Shen L , Zhong Y , Li Z , Liu Z , Li Q . 2022 Species associations and conservation of sichuansnub-nosed monkey in the tangjiahe. Nat. Nat. Res. Chin. J. Wildl. **44** , 1–13. (10.12375/ysdwxb.20230101)

[27] Su T . 2022 Study of diversity and spatial of mammals and land fowls in gansu liancheng national nature reserve, china. Ph.D. Thesis, [Beijing, China]: Beijing Forestry University.

[28] Shen L , Gao Z , Ou W , Chen W , Ma W . 1999 Surveys of amphibians and reptiles in Tangjiahe nature reserve, Sichuan province. Sichuan J. Zool. **18** , 3–5. (10.3969/j.issn.1000-7083.1999.03.016)

[29] Yao G , Li Y , Zhang J , Li D , Yang Z , Hu J , Shi X . 2017 An investigation on population density and distribution of Rusa unicolor in Wolong national nature Reserve. Sichuan J. Zool. **36** , 588–592. (10.11984/j.issn.1000-7083.20170036)

[30] Wen X , Yan Y , He M , Ran J , Ma X , Qiu R , Wang N . 2020 A survey on the population densities of three Galliformes species by using two different methods in the Heizhugou national nature reserve. Sichuan. Sichuan J. Zool. **39** , 68–74. (10.11984/j.issn.1000-7083.20190036)

[31] Zhang R . 1999 Chapter 5: zoological geographical division. In Zoogeography of china (ed. H Zhu ), pp. 131–132. Beijing: Science Press.

[32] Sandel B , Arge L , Dalsgaard B , Davies RG , Gaston KJ , Sutherland WJ , Svenning JC . 2011 The influence of late quaternary climate-change velocity on species endemism. Science **334** , 660–664. (10.1126/science.1210173) 21979937

[33] Smith AT , Xie Y . 2009 A guide to the mammals of China. (eds Y Chen , R Yang , J Zhang ). Changsha: Hunan Education Publishing House.

[34] Mackinnon J , Phillipps K , He F . 2019 A field guide to the birds of china. (eds H Lu , F He ). Changsha: Hunan Education Publishing House.

[35] Siegel S , Castellan J . 1988 Chapture 9: measures of association and their tests of significance. In Nonparametric statistics for the behavioral sciences, second edition. New York: McGraw-Hill International Editions.

[36] Blanchet FG , Cazelles K , Gravel D . 2020 Co-occurrence is not evidence of ecological interactions. Ecol. Lett. **23** , 1050–1063. (10.1111/ele.13525) 32429003

[37] Forbes SA . 1907 On the local distribution of certain illinois fishes: an essay in statistical ecology. Ill. State Lab. Nat. Hist. **7** , 273–303. (10.21900/j.inhs.v7.407)

[38] Abramov AV . 2016 Meles leucurus. the IUCN red list of threatened species 2016: e.T136385A45221149.

[39] Zhang L , Wang A , Yuan L , Bao W , Yang Y , Ba T . 2011 Preliminary comparison of diet composition of four small sized carnivores at Saihanwula nature reserve, inner Mongolia. Acta. Theriol. Sin. **31** , 55–61. (10.16829/j.slxb.2011.01.009)

[40] Yi X . 2005 Food resource partitioning in Altai Weasel, Steppe Polecat and upland buzzard : evidence from stable Isotope ratios. Zool. Res. **26** , 1–7. (10.3321/j.issn:0254-5853.2005.01.001)

[41] Gao Y , Wang S , Zhang M , Ye Z , Zhou J . 1987 Vol.8: Carnivora. In Zoology of China: mammalia (ed. Y Gao ), pp. 323–332. Beijing: Science Press.

[42] Abramov AV . 2016b Mustela altaica. the IUCN red list of threatened species 2016 e.T41653A45213647.

[43] Santos MJ , Santos-Reis M . 2010 Stone marten (Martes foina) habitat in a mediterranean ecosystem: effects of scale, sex, and interspecific interactions. Eur. J. Wildl. Res. **56** , 275–286. (10.1007/s10344-009-0317-9)

[44] Wei W , Zhou W , Fan N , Biggins DE . 1994 Habitat selection, feeding and caring for the young of altai weasel. Acta. Theriol. Sin. **14** , 184–188. (doi:10.16829/j .slxb.1994.03.005)

[45] Martinoli A , Preatoni D . 1995 Food habits of the stone marten (mares foina) in the upper aveto valley (northern apennines, italy). Hystrix, (n.s.). **7** , 137–142. (10.4404/hystrix-7.1-2-4062)

[46] Bakaloudis DE , Vlachos CG , Papakosta MA , Bontzorlos VA , Chatzinikos EN . 2012 Diet composition and feeding strategies of the stone Marten (Martes foina) in a typical mediterranean ecosystem. Sci. World J. **2012** , 163920. (10.1100/2012/163920) PMC3349108 22619607

[47] Zeng G , Zheng H , Deng T . 2009 Summer cave selection of hog badger (Arctonyx collaris) on the North slope of Funiu mountain. Acta Ecol. Sin. **29** , 208–215. (10.3321/j.issn:1000-0933.2009.01.026)

[48] Zhu B , Wang B , Ran J , Li B , Huang F , Li X , Gu X . 2019 Seasonal variation of daily activity patterns and diet of yellow-throated Marten (Martes flavigula). Acta. Theriol. Sin. **39** , 52–61. (doi:10. 16829 /j. slxb. 150178)

[49] Wang H , Fuller TK . 2003 Food habits of four sympatric carnivores in southeastern China. Mammalia **67** , 513–519. (10.1515/mamm-2003-0405)

[50] Zhou YB , Newman C , Buesching CD , Zalewski A , Kaneko Y , Macdonald DW , Xie ZQ . 2011 Diet of an opportunistically frugivorous carnivore, Martes flavigula, in subtropical forest. J. Mammal. **92** , 611–619. (10.1644/10-MAMM-A-296.1)

[51] Zhou Y , Chen W , Kaneko Y , Newman C , Liao Z , Zhu X , Buesching CD , Xie Z , Macdonald DW . 2015 Seasonal dietary shifts and food resource exploitation by the hog-badger (Arctonyx collaris) in a Chinese subtropical forest. Eur. J. Wildl. Res. **61** , 125–133. (10.1007/s10344-014-0881-5)

[52] Zhou YB , Slade E , Newman C , Wang XM , Zhang SY . 2008 Frugivory and seed dispersal by the yellow-throated marten, Martes flavigula, in a Subtropical forest of China. J. Trop. Ecol. **24** , 219–223. (10.1017/S0266467408004793)

[53] Li N , Zhong M , Leng X , Wan A , Fang S , An S . 2015 Seed dispersal effectiveness of plant by frugivores: a review. Chin. J. Ecol. **34** , 2041–2047. (10.13292/j.1000-4890.20150616.001)

[54] Abramov AV , Kranz A , Herrero J , Choudhury A , Maran T . 2016 Martes foina. The IUCN red list of threatened species 2016: e.T29672A45202514.

[55] Liu Z , Zhang L , Zhou H , Li Q , Wang X , Shi X , Li Z . 2022 Effects of human interferences on the diversity of terrestrial bird and mammal in Wolong national nature reserve, China. Chin. J. Wildl. **43** , 897–906. (10.12375/ysdwxb.20220404)

[56] Fossilworks. See https://fossilworks.org (accessed 23 October 2023).

[57] Cai Y , Li B . 2018 Advances of mitochondrial genome and phylogenesis in guloninae. Chin. J. Wildl. **39** , 426–432. (10.19711/j.cnki.issn2310-1490.2018.02.038)

[58] Donadio E , Buskirk SW . 2006 Diet, morphology, and interspecific killing in carnivora. Am. Nat. **167** , 524–536. (10.1086/501033) 16670995

[59] Li G . 1987 Martes foina. In Fauna sinica: mammalia, pp. 128–129, vol. 8. Beijing: Science Press.

[60] Ritchie EG , Johnson CN . 2009 Predator interactions, mesopredator release and biodiversity conservation. Ecol. Lett. **12** , 982–998. (10.1111/j.1461-0248.2009.01347.x) 19614756

[61] Duckworth JW , Timmins R , Chutipong W , Gray TNE , Long B , Helgen K , Rahman H , Choudhury A , Willcox DHA . 2016 Arctonyx collaris. The IUCN red list of threatened species 2016: e.T70205537A45209459. (10.2305/IUCN.UK.2016-1.RLTS.T70205537A45209459.en)

[62] Proulx G *et al* . 2004 World distribution and status of the genus *Martes* in 2000. In Martens and fishers (Martes) in human-altered environments (eds DJ Harrison , AK Fuller , G Proulx ), pp. 21–76. New York, NY: Springer. (10.1007/b99487)

[63] Ricklefs RE . 1990 Ecology, Third Edition. New York: W. H. Freeman and Company.

[64] Han L . 2006 Paleolithic culture evolution and its relationship with environmental changes in Anhui Province, China. In Proceedings of the Tenth Annual meeting of the Chinese Society of Vertebrate Paleontology, pp. 155–162. Beijing: China Ocean Press.

[65] Cox CB , Moore PD , Ladle RJ . 2016 Biogeography: an ecological and evolutionary approach, Ninth Edition. Wiley Blackwell.

[66] Dong G , Chai Z , Chen H *et al* . 2002 Chapter 4: the evolution of ecological environment (lower part. In Assessments on the environment evolution in Western China: Volumn 1: the environmental characteristics of the Western China and their evolution (eds S Wang , G Dong ), pp. 104–144. Beijing, China: Science Press.

[67] Liu Z , Su T , Li Q , Li Z . 2024 Spatial distribution pattern of mustelids in the eastern edge of the qinghai-tibet plateau [dataset]. Dryad (10.5061/dryad.7m0cfxq2w) PMC11303035 39113774

